# Anterograde Trafficking of KCa3.1 in Polarized Epithelia Is Rab1- and Rab8-Dependent and Recycling Endosome-Independent

**DOI:** 10.1371/journal.pone.0092013

**Published:** 2014-03-14

**Authors:** Claudia A. Bertuccio, Shih-Liang Lee, Guangyu Wu, Michael B. Butterworth, Kirk L. Hamilton, Daniel C. Devor

**Affiliations:** 1 Department of Cell Biology, School of Medicine, University of Pittsburgh, Pittsburgh, Pennsylvania, United States of America; 2 Department of Physiology, Otago School of Medical Sciences, University of Otago, Dunedin, Otago, New Zealand; 3 Department of Pharmacology and Toxicology, Medical College of Georgia, Georgia Regents University, Augusta, Georgia, United States of America; Cinvestav-IPN, Mexico

## Abstract

The intermediate conductance, Ca^2+^-activated K^+^ channel (KCa3.1) targets to the basolateral (BL) membrane in polarized epithelia where it plays a key role in transepithelial ion transport. However, there are no studies defining the anterograde and retrograde trafficking of KCa3.1 in polarized epithelia. Herein, we utilize Biotin Ligase Acceptor Peptide (BLAP)-tagged KCa3.1 to address these trafficking steps in polarized epithelia, using MDCK, Caco-2 and FRT cells. We demonstrate that KCa3.1 is exclusively targeted to the BL membrane in these cells when grown on filter supports. Following endocytosis, KCa3.1 degradation is prevented by inhibition of lysosomal/proteosomal pathways. Further, the ubiquitylation of KCa3.1 is increased following endocytosis from the BL membrane and PR-619, a deubiquitylase inhibitor, prevents degradation, indicating KCa3.1 is targeted for degradation by ubiquitylation. We demonstrate that KCa3.1 is targeted to the BL membrane in polarized LLC-PK_1_ cells which lack the μ1B subunit of the AP-1 complex, indicating BL targeting of KCa3.1 is independent of μ1B. As Rabs 1, 2, 6 and 8 play roles in ER/Golgi exit and trafficking of proteins to the BL membrane, we evaluated the role of these Rabs in the trafficking of KCa3.1. In the presence of dominant negative Rab1 or Rab8, KCa3.1 cell surface expression was significantly reduced, whereas Rabs 2 and 6 had no effect. We also co-immunoprecipitated KCa3.1 with both Rab1 and Rab8. These results suggest these Rabs are necessary for the anterograde trafficking of KCa3.1. Finally, we determined whether KCa3.1 traffics directly to the BL membrane or through recycling endosomes in MDCK cells. For these studies, we used either recycling endosome ablation or dominant negative RME-1 constructs and determined that KCa3.1 is trafficked directly to the BL membrane rather than via recycling endosomes. These results are the first to describe the anterograde and retrograde trafficking of KCa3.1 in polarized epithelia cells.

## Introduction

In various epithelia, including colonic, airway and salivary epithelia, agonist-mediated activation of Ca^2+^-dependent K^+^ channels (KCa) is known to play a key role in the regulation of transepithelial ion and water transport. Thus, transepithelial Cl^−^ secretion requires activation of numerous transporters/channels, including the Na^+^/K^+^-ATPase on the basolateral (BL) membrane to maintain transmembrane ionic gradients. Also, activation of the BL membrane Na^+^-K^+^-2Cl^−^ cotransporter allows Cl^−^ to accumulate above its electrochemical equilibrium. Activation of an apical membrane Cl^−^ channel allows Cl^−^ to move down its equilibrium potential. Finally, activation of BL membrane K^+^ channels maintains a membrane potential favorable for the continuous Cl^−^ efflux across the apical membrane, while also recycling K^+^ taken up by Na^+^-K^+^-2Cl^−^ cotransporter and the Na^+^/K^+^-ATPase. We previously characterized the KCa in colonic epithelia using both whole-cell [1] and single channel [2] methods and later confirmed this was KCa3.1 [3] following its molecular cloning [4], [5]. It is now well-recognized that KCa3.1 is a major BL K^+^ channel critical for maintenance of the electrochemical driving force for Ca^2+^-mediated Cl^−^ secretion across these epithelia [6], [7], [8], [9]. Given the critical role of KCa3.1 in maintaining transepithelial ion and fluid transport, it is not surprising that this channel has been suggested to play a role in the etiology of various diseases. Indeed, KCa3.1 has been implicated in Crohn's disease [10], ulcerative colitis [11], cystic fibrosis and chronic obstructive pulmonary disease [12], [13] and ADPKD cyst formation [14].

Clearly, a key component dictating the physiological response of an epithelial cell to an increase in Ca^2+^ is the number of KCa3.1 channels at the plasma membrane. We [15], [16], [17] and others [18] have identified molecular motifs within the N- and C-termini, as well as the transmembrane domains, that are critical in the assembly and anterograde trafficking of KCa3.1. Utilizing a Biotin Ligase Acceptor Peptide (BLAP)-tagged KCa3.1 we demonstrated, in human embryonic kidney (HEK293) cells and human microvascular endothelial (HMEC-1) cells, that KCa3.1 is endocytosed from the plasma membrane and targeted to the lysosome via an endosomal complex required for transport (ESCRT)- and Rab7-dependent pathway [19]. Further, we demonstrated that KCa3.1 is initially ubiquitylated following endocytosis and then deubiquitylated by USP8 prior to lysosomal degradation [20]. Schwab and colleagues [21] have also demonstrated that KCa3.1 is endogenously expressed in MDCK cells and that it is endocytosed in a clathrin-dependent manner in non-polarized, migrating cells.

In contrast to the studies above, there is little information regarding the anterograde and retrograde trafficking of KCa3.1 in polarized epithelia. Therefore, the aim of this study was to investigate the trafficking of KCa3.1 in polarized epithelia. Herein, we demonstrate that KCa3.1 is expressed solely at the BL membrane in the model polarized epithelial cell lines, MDCK, Caco-2, FRT and LLC-PK_1_, indicating this localization is independent of the adaptor protein, μ1B. In polarized cells, KCa3.1 is ubiquitylated at the BL membrane and this is increased following endocytosis after which the channel is targeted for protosomal and lysosomal degradation. We further demonstrate that Rab1 and Rab8 are critical for ER/Golgi exit and subsequent plasma membrane expression of KCa3.1. Finally, we demonstrate that, following Golgi exit, KCa3.1 does not traffic through either RME-1- or transferrin receptor (TfnR)-positive recycling endosomes on the way to the plasma membrane in polarized epithelia, suggesting KCa3.1 traffics directly to the plasma membrane. These results are the first to characterize the anterograde and retrograde trafficking of KCa3.1 in polarized epithelia cells.

## Materials and Methods

### Molecular Biology

Our HA-, myc- and biotin ligase acceptor peptide (BLAP)-tagged KCa3.1 (also referred to as IK1 or SK4) constructs, as well as the bicistronic plasmid (pBudCE_4.1_) expressing both BLAP-KCa3.1 and BirA-KDEL have been previously described [22], [23], [24]. BLAP-KCa3.1 was also subcloned in to the pAdlox (SwaI modified) adenoviral shuttle plasmid using EcoRI and SalI restriction sites. In order to biotinylate KCa3.1 within the endoplasmic reticulum before trafficking to the plasma membrane, we subcloned BLAP-KCa3.1 as well as BirA-KDEL (generously provided by Dr. Alice Ting, Massachusetts Institute of Technology, Cambridge, MA) into the bicistronic adenoviral shuttle plasmid DUALCCM-CMW-MCS2 (Vector Biolabs; Philadelphia, PA). In this construct, both cDNAs are located behind unique CMV promoters. BLAP-KCa3.1 and BirA-KDEL were sequentially subcloned using EcoRI/XhoI and NheI/SalI restriction sites, respectively. BLAP-KCa3.1 and BirA-KDEL/BLAP-KCa3.1 replication deficient adenoviruses were generated by the University of Pittsburgh Vector Core facility and Vector Biolabs, respectively. The transferrin receptor (TfnR) adenovirus was generously provided by Dr. Ora Weisz (University of Pittsburgh, Pittsburgh, PA). Generation of the GFP- and Flag-tagged variants of Rabs 1, 2, 6 and 8 have been previously described [25], [26], [27]. Rab 10 was purchased from Addgene (Cambridge, MA). Wild type (WT) and dominant negative (DN) GFP-tagged receptor-mediated endocytosis-1 (RME-1) constructs were generously provided by Dr. Barth Grant (Rutgers University, New Brunswick, NJ).

### Cell Culture

Madin-Darby canine kidney (MDCK) and pig epithelial (LLC-PK_1_) cells were cultured in α-MEM medium, human epithelial colorectal adenocarcinoma (Caco-2) and human embryonic kidney (HEK293) cells were cultured in Dulbecco's modified Eagle's medium (Invitrogen) and Fischer rat thyroid (FRT) epithelial cells were grown in F12 (Sigma-Aldrich, St. Louis, MO). All media were supplemented with 10% fetal calf serum, and 1% penicillin/streptomycin. Both the wild type LLC-PK_1_ cells and the LLC-PK_1_ cell line stably expressing the FLAG-tagged μ1B subunit of the AP1 adaptor complex was generously provided by Dr. Michael Caplan (Yale University, New Haven, CT) [28]. The MDCK, Caco-2, HEK293, LLC-PK_1_ and FRT cell lines were obtained from the ATCC (Manassas, VA). All cells were grown in a humidified 5% CO_2_/95% O_2_ incubator at 37°C. A stable FRT cell line was generated by transfecting in the pBudCE_4.1_ bicistronic plasmid expressing both BLAP-KCa3.1 and BirA-KDEL using Lipofectamine 2000 (Invitrogen) following the manufacturer's instructions and selecting a stable cell line using zeocin (850 μg/ml) (protocol approved by the University of Otago Institutional Biological Safety Committee, approval code GMD003298-33).

HEK293 cells were transfected using Lipofectamine 2000 following the manufacturer's instructions. MDCK, Caco-2, LLC-PK_1_ and FRT cells were seeded onto Transwell® permeable supports (Corning Inc., Corning, NY) and grown to confluence forming a polarized epithelium (3–4 days post seeding). Polarized MDCK, Caco-2, LLC-PK_1_ or FRT cells were transduced with the BLAP-KCa3.1 or BirA-KDEL/BLAP-KCa3.1 adenoviruses, as indicated for each experiment. Briefly, well-polarized cells were washed three times with Ca^2+^-free PBS followed by addition of adenovirus to the apical side of the filter. After 1 hr of incubation at 37°C in a 5% CO_2_/95% O_2_ atmosphere, cells were washed once with PBS and allowed to recover until the next day in normal growth media. In some experiments, 6 hrs after adenoviral addition in MDCK cells the cells were further transfected with WT or DN RME-1 or appropriate Rab construct using Lipofectamine 2000, following the manufacturer's instructions.

### Biotinylation of KCa3.1 using recombinant biotin ligase

BLAP-tagged KCa3.1 was either biotinylated using recombinant biotin ligase (BirA), as described [22] or by co-expression with BirA-KDEL [23]. Plasma membrane BLAP-KCa3.1 was then labeled with streptavidin-Alexa555 (0.01 mg/ml, Invitrogen) for 15 min at 4°C. The cells were extensively washed to remove unbound streptavidin and incubated for various periods of time at 37°C as indicated or immediately fixed and permeabilized with 2% paraformaldehyde plus 0.1% Triton X-100 [22]. The apical plasma membrane was labeled with WGA-Alexa488 (wheat germ agglutinin, 5 μg/ml), (Invitrogen). Nuclei were labeled with DAPI (Sigma-Aldrich). Cells were imaged by laser-scanning confocal microscopy (Olympus FluoView 1000 system) using a 63× oil immersion lens as described [19]. Z-stacks were taken to cover the entire thickness of the cell in a step size of 0.5 μm.

### Antibodies

GFP antibody was obtained from Santa Cruz Biotechnology, Inc. (Santa Cruz, CA). Monoclonal α-streptavidin antibody was obtained from Abcam (Cambridge, MA). Monoclonal Flag, α-tubulin and β-actin were purchased from Sigma-Aldrich. Antibodies against Myc and HA were obtained from Covance (Princeton, NJ) and Rab8 antibody was purchased from BD Transduction laboratories (San Jose, CA).

### Immunoprecipitations and immunoblots

Our immunoprecipitation (IP) and immunoblot (IB) protocols have been described [15], [16]. Briefly, cells were lysed and equivalent amounts of total protein were pre-cleared with protein G-agarose beads (Invitrogen) followed by incubation with the indicated antibody. Normal IgG was used as negative control. Immune complexes were precipitated with protein G-agarose beads, washed extensively, and resuspended in Laemmli sample buffer. Proteins were resolved by SDS-PAGE and transferred to nitrocellulose for IB analysis. To eliminate interference by the heavy and light chains of the immunoprecipitating antibody in the IP, mouse or rabbit IgG Trueblot ULTRA (eBioscience, San Diego, CA) were used as a secondary antibody for the detection of immunoprecipitated proteins in the IB.

### Determination of degradation rate for plasma membrane of KCa3.1

The degradation rate for endocytosed membrane KCa3.1 was determined as described [22]. Briefly, KCa3.1 in polarized MDCK, Caco-2 or FRT cells was specifically biotinylated using BirA and labeled with non-conjugated streptavidin after which the cells were incubated for various periods of time at 37°C, as indicated. In some experiments, the lysosomal protease inhibitors leupeptin (100 μM) and pepstatin (1 μg/ml; Leu/Pep) (Sigma-Aldrich), the proteasome inhibitor lactacystin (10 μM, Lacta) or a general deubiquitylase (DUB) inhibitor PR-619 (50 μM) (LifeSensors Inc., Malvern, PA) were added to both apical and BL membranes prior to the 37°C incubation step. The cells were then lysed and equivalent amounts of total protein were separated by SDS-PAGE, followed by IB for streptavidin. Bands were quantified by densitometry using ImageJ software (NIH; http://rsb.info.nih.gov/ij/). The obtained band intensities for the various time points were normalized relative to the intensity at time 0 (T = 0) and are reported. The blots were also probed for α-tubulin and β-actin as a protein-loading control.

### Pulldown of ubiquitylated KCa3.1 using TUBEs

To determine the ubiquitylation state of KCa3.1 at the plasma membrane, and following endocytosis, we used GST-tagged tandem-repeat ubiquitin-binding entities (TUBEs) (LifeSensors Inc.), as described [20]. Caco-2 cells were enzymatically biotinylated and streptavidin labeled at the plasma membrane after which the cells were lysed in the presence of GST-TUBEs (200 μg/ml) or returned to 37°C for various periods of time to allow endocytosis to occur, and then lysed in the presence of TUBEs. The TUBEs were subsequently pulled down on glutathione agarose beads followed by SDS-PAGE. The resulting IB was probed with α-streptavidin Ab. In this way, only the streptavidin-tagged KCa3.1, which was ubiquitylated and hence bound to TUBEs, was detected [20].

### Recycling endosome ablation assay

MDCK cells were transduced with TfnR and BirA-KDEL/BLAP-KCa3.1 adenoviruses. After 24 hrs, recycling endosome ablation was carried out based on the methods of Ang et al. [29]. MDCK cells were incubated with 0.010 mg/ml Tfn-HRP and Tfn-Alexa488 in serum starved media for 45 min at 37°C, washed twice in serum-free αMEM media, and incubated for 20 min at 37°C. Cells were then washed twice on ice-cold PBS. Surface-bound Tfn-HRP and Tfn-alexa488 were removed by two 5 min washes in 0.15 M NaCl plus 20 mM citric acid, pH 5.0. After washes with ice-cold PBS, the cells were incubated in PBS containing 0.1 mg/ml diaminobenzidine (DAB; Sigma-Aldrich) in the absence (control) or presence (ablation) of H_2_O_2_ (0.025%) for 1 hr on ice in the dark. The Tfn-HRP reacts with DAB and H_2_O_2_ forming an insoluble precipitate that prevents the fusion of post-Golgi vesicles with the recycling endosome. The ablation reaction was stopped by washing cells twice in PBS with 1% BSA. Following recycling endosome ablation, neutravidin biotin binding protein (600 μg/ml) (Thermo Scientific, Rockford, IL) was added to the control and ablated cells for 2 hrs at 4°C such that all plasma membrane localized BLAP-KCa3.1 channels would be bound and “blocked” from binding to subsequently added streptavidin. The cells were then incubated in media with cyclohexamide (400 μg/ml, Sigma-Aldrich) for 90 min at 37°C to allow ER/Golgi-resident KCa3.1 channels to potentially traffic to the plasma membrane. To determine whether ER/Golgi-resident channels had trafficked to the plasma membrane following recycling endosome ablation we labeled with streptavidin-Alexa555 (0.01 mg/ml, Invitrogen) for 15 min at 4°C.

### KCa3.1 trafficking from Golgi to the plasma membrane

MDCK cells were transduced with BirA-KDEL/BLAP-KCa3.1 adenovirus and subsequently transfected with GFP-tagged WT or DN RME-1. Plasma membrane localized and biotinylated BLAP-KCa3.1 was “blocked” with neutravidin (600 μg/ml), as above. In order to allow accumulation of channels in the Golgi, cells were incubated for 2 hrs at 19°C in the continued presence of neutravidin. Cells were then washed twice on ice-cold PBS, warm media was added and the cells were incubated for 30 min or 2 hrs at 37°C to allow channels to traffic to the plasma membrane. Finally, the newly resident plasma membrane channels were labeled with streptavidin-Alexa555, for IF localization studies or with non-conjugated streptavidin followed by IB with α-streptavidin antibody to quantify the rate of plasma membrane KCa3.1 appearance. Similarly, HEK293 cells were transfected with BirA-KDEL/BLAP-KCa3.1 and each Rab construct followed by neutravidin “block”, incubation at 19°C to allow KCa3.1 Golgi accumulation and subsequent incubation at 37°C for the indicated periods of time to allow trafficking of KCa3.1 from the ER/Golgi to the plasma membrane. Plasma membrane localized KCa3.1 was then labeled with non-conjugated streptavidin, followed by IB with α-streptavidin antibody to quantify plasma membrane appearance.

### Statistical analysis

All data are presented as means ± SEM, where **n** indicates the number of experiments. Statistical analysis was performed using a Student's ***t***-test. To compare the normalized values of the IB band intensities, statistical analysis was performed using the non-parametric Kruskal-Wallis test. The traffic from ER/Golgi to plasma membrane was analyzed using a two-way Anova test followed by Bonferroni post-test comparing WT and DN at each time point. A value of p<0.05 is considered statistically significant and is reported.

## Results

### Expression and localization of KCa3.1 in polarized epithelial cells

**Figure 1:**
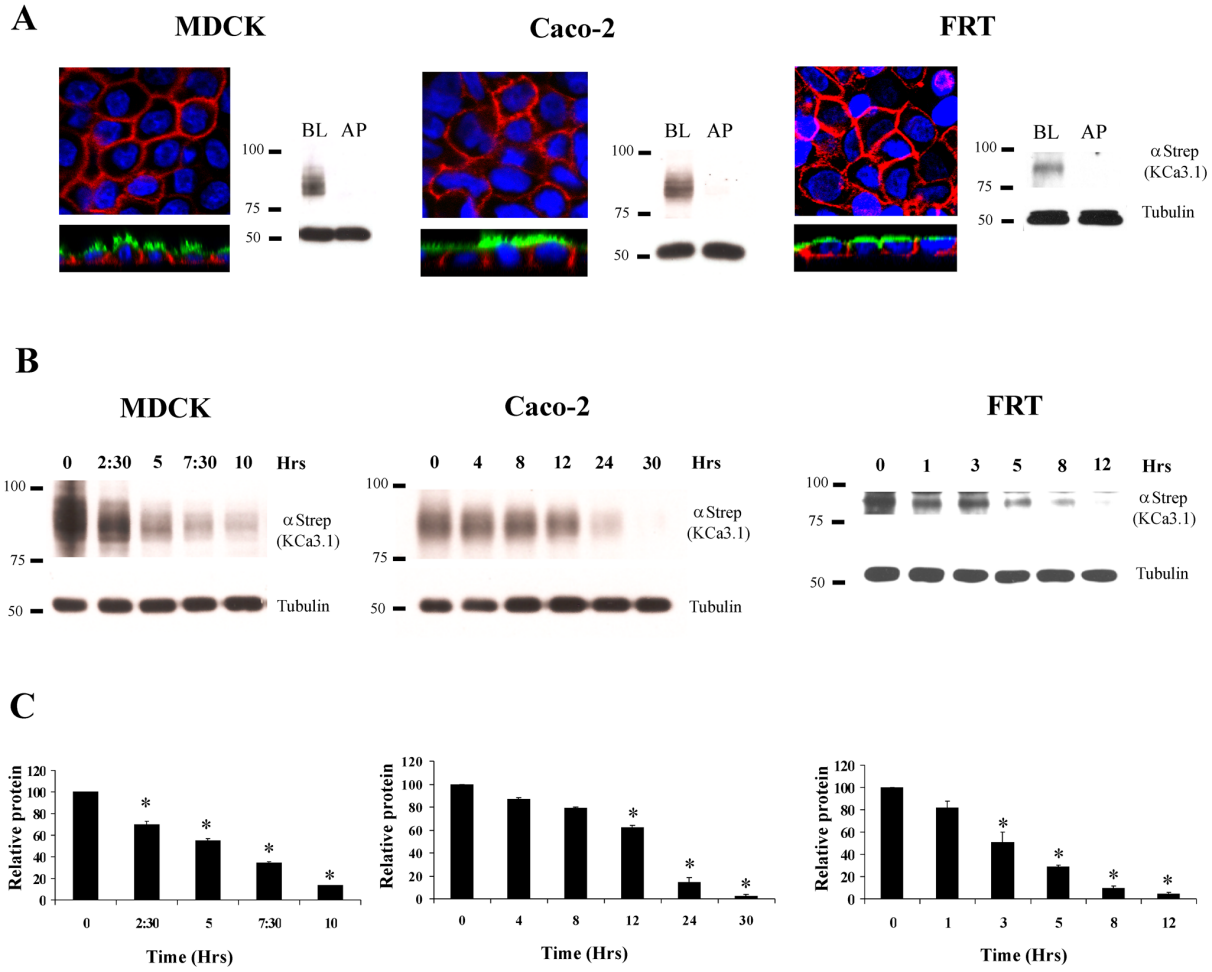
Localization and degradation of KCa3.1 in polarized epithelia cells. **A**. BLAP-KCa3.1 was transduced into MDCK, Caco-2 or FRT cells grown to confluence on Transwell® filters. Basolateral (BL) and Apical (AP) plasma membrane KCa3.1 channels were specifically biotinylated using recombinant BirA and labeled with either streptavidin-Alexa555 (red) for IF localization or unconjugated streptavidin for IB. Apical membrane was co-labeled with wheat germ agglutinin (WGA)-Alexa488 (green) and nuclei were labeled with DAPI (blue) for IF localization. The top panels show a single confocal section through the mid-plane of the cells and the bottom panels show a z-stack. For the IB experiments, BL and AP membranes were independently labeled on separate Transwell filters. 30 μg of protein was loaded per lane. Data are representative of 3 separate experiments. KCa3.1 was localized exclusively to the BL membrane as assessed by both IF and IB. **B**. KCa3.1 was biotinylated at the BL membrane as above in MDCK and Caco-2 cells or biotinylated at the level of the ER in FRT cells stably expressing BirA-KDEl/KCa3.1-BLAP and its degradation evaluated over time. Note the different time points used for each cell line. Tubulin was used as a loading control. **C**. Quantification of the blots shown in B for at least 3 separate experiments. *P<0.05 with respect to T = 0.

To determine the plasma membrane localization of KCa3.1 in polarized epithelial cells, we transduced MDCK, Caco-2 and FRT cells grown on Transwell filters with BLAP-KCa3.1 adenovirus. The following day, KCa3.1 was labeled specifically at the plasma membrane using recombinant BirA followed by incubation with streptavidin-Alexa555. The apical plasma membrane was co-labeled with WGA–Alexa488. As shown in Fig. 1A, BLAP-KCa3.1 was localized exclusively to the basolateral (BL) membrane of each of these polarized epithelial cells. These results were confirmed using Transwell®-grown cells, which were independently labeled at either the apical (AP) or BL membrane with streptavidin followed by IB using an α-streptavidin antibody. As is apparent, KCa3.1 is highly expressed at the BL membrane with negligible expression associated with the apical membrane (Fig. 1A). To determine the degradation time of KCa3.1 at the BL membrane we labeled channels and allowed internalization at 37°C for the indicated periods of time followed by IB, as above. As shown in Figs. 1B and C, KCa3.1 was degraded with a similar time-course in MDCK and FRT cells, whereas in Caco-2 cells the degradation rate was slowed.

### Basolateral membrane KCa3.1 is targeted to the lysosome and proteasome for degradation

**Figure 2:**
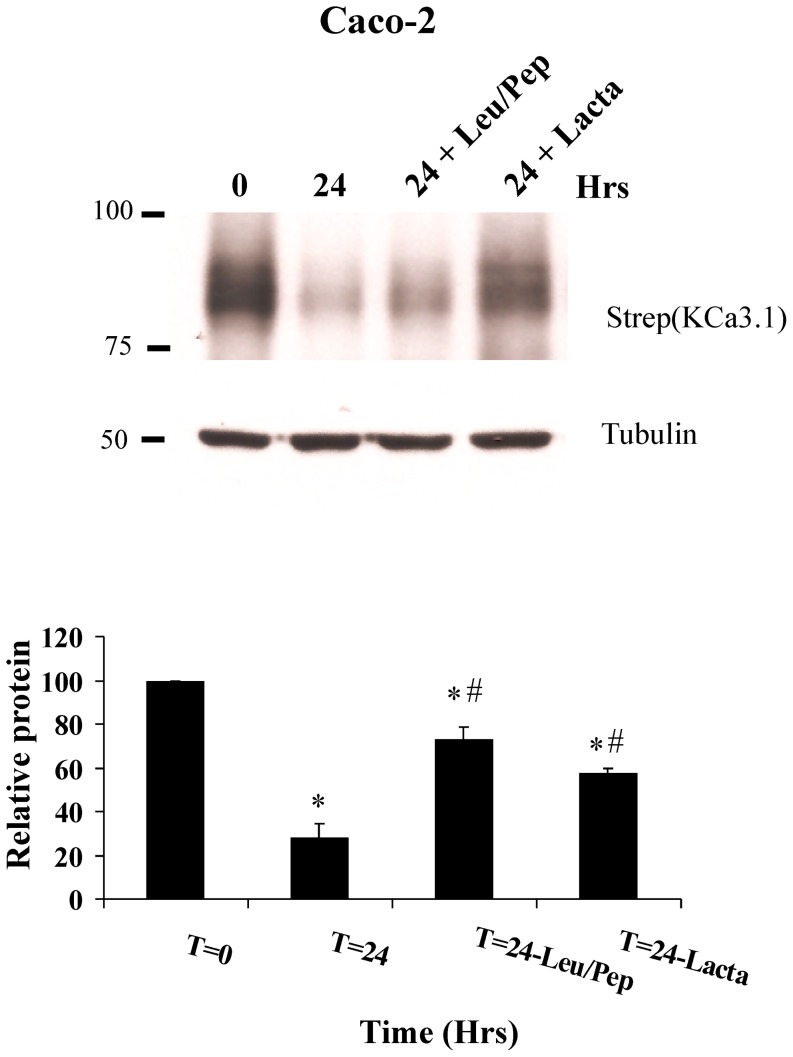
BL localized KCa3.1 is degraded in the lysosome. BLAP-KCa3.1 was transduced in Caco-2 cells grown to confluence on Transwell® filters and BL plasma membrane channels specifically biotinylated and labeled with streptavidin. Cells were treated with leupeptin (100 μM) and pepstatin (1 μg/ml) (Leu/Pep) or lactacystin (10 μM, Lacta) and degradation of KCa3.1 determined. Both Leu/Pep and Lacta inhibited degradation of KCa3.1. 30 μg of protein was loaded per lane. Averages from 3 experiments are plotted below; *P<0.05 with respect to T = 0, # P< 0.05 with respect to T = 24 hrs in Caco-2 cells.

To determine whether lysosomes are implicated in the degradation of BL membrane KCa3.1 we utilized Caco-2 and FRT cells grown on Transwell® filters under normal conditions and following inhibition of lysosomal proteases with leupeptin and pepstatin (Leu/Pep). BLAP-KCa3.1 was biotinylated and labeled with streptavidin, as above and incubated at 37°C for the times indicated followed by IB to determine the amount of KCa3.1 remaining. As shown in Fig. 2, in Caco-2 cells 28±3% of KCa3.1 remained after 24 hrs and this was increased to 73±6% in the presence of Leu/Pep (n = 4; P<0.05). Also, in FRT cells we observed a similar effect of Leu/Pep at 5 hrs, i.e., in control cells 29±1% of KCa3.1 remained and this was increased to 81±4% (n = 6, P<0.05) in the presence of Leu/Pep (data not shown), indicative of lysosomal degradation following endocytosis.

We also determined whether the proteasome inhibitor, lactacystin would alter the degradative fate of BL KCa3.1 expressed in polarized epithelia. As shown in Fig. 2, lactacystin abrogated KCa3.1 degradation such that 67±2% of KCa3.1 remained in Caco-2 cells after 24 hrs; significantly more than in control conditions (n = 4; P<0.05), suggesting a role for the proteasome in the degradation of BL KCa3.1.

### KCa3.1 is ubiquitylated at the plasma membrane and following endocytosis

**Figure 3:**
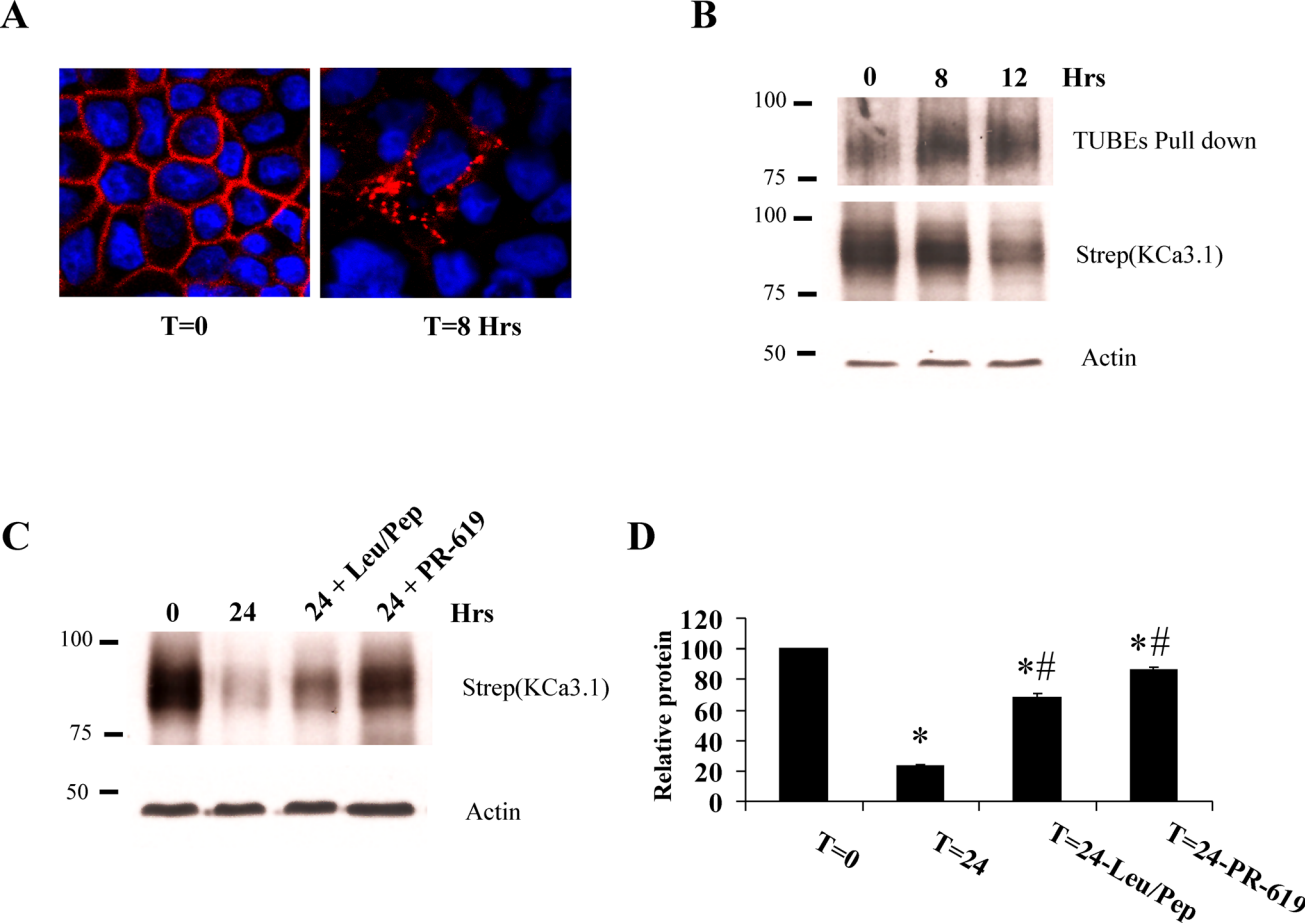
KCa3.1 is ubiquitylated following endocytosis in polarized Caco-2 cells. A. IF localization of KCa3.1 (red) in confluent Caco-2 cells on Transwell® filters following labeling as in Fig. 1 at T = 0 and following 8 hrs at 37°C. After 8 hrs, KCa3.1 is endocytosed. Nuclei are labeled with DAPI (blue). **B**. Ubiquitylated KCa3.1 was evaluated by TUBEs pulldown at T = 0 when KCa3.1 is localized to the BL membrane and following endocytosis for 8 and 12 hrs (see Methods). An increase in ubiquitylated KCa3.1 is apparent at 8 and 12 hrs despite the channel being degraded [strep(KCa3.1) blot]. Actin was used as a loading control. **C**. Inhibition of deubiquitylases by PR-619 (50 μM) prevents degradation of KCa3.1. Leu/Pep was used as a control for these experiments. 30 μg of protein was loaded per lane. Actin was used as a loading control. **D**. Quantification of the blots shown in C for at least 3 separate experiments; *P<0.05 with respect to T = 0, # P<0.05 with respect to T = 24 hrs.

To determine whether KCa3.1 is ubiquitylated in polarized epithelia, Caco-2 cells were grown on Transwell® supports and KCa3.1 expressed and labeled, as above. As shown in Fig. 3A, at T = 0, KCa3.1 is localized to the BL membrane and after 8 hrs at 37°C, KCa3.1 has been endocytosed. To evaluate the ubiquitylation of KCa3.1, cells were lysed in the presence of GST-tagged TUBEs (see Methods) [20]. At T = 0, we observed a small amount of ubiquitylated KCa3.1 (Fig. 3B, lane 1) and this was significantly increased following endocytosis for 8 or 12 hrs (8 hrs: 143±4% and 12 hrs: 139±1%; n = 3, P<0.05, Fig. 3B, lane 2 and 3), despite the decreased total expression of KCa3.1 due to degradation. These results indicate that KCa3.1 is ubiquitylated at the BL membrane of polarized epithelia and this is increased following endocytosis.

Deubiquitinating enzymes (DUBs) are proteases that remodel or cleave ubiquitin from target proteins before they undergo proteasome-and/or lysosome-degradation [30], [31]. We determined whether DUBs play a role in KCa3.1 degradation in polarized Caco-2 cells, transduced with BLAP-KCa3.1 and grown on Transwell® supports, as above. The cells were incubated at 37°C for 24 hrs in the presence or absence of the general DUB inhibitor, PR-619 after which the expression of KCa3.1-BLAP was evaluated by IB. As shown in Fig. 3C and D, after 24 hrs KCa3.1 was extensively degraded, averaging 24±1% of that observed at T = 0 (n = 3; P<0.05), similar to our results above. In PR-619 treated cells, channel degradation was significantly inhibited with 87±1% of KCa3.1 still remaining after 24 hrs (n = 3; P<0.05). The lysosomal protease inhibitors Leu/Pep were used as a positive control in these experiments and prevented degradation of KCa3.1, similar to our results above with 68±2.6% of KCa3.1 remaining (P<0.05). These results suggest that the deubiquitylation of KCa3.1 by DUBs is required for proper degradation of the channel in polarized epithelia.

### Trafficking of KCa3.1 from the ER/Golgi to the plasma membrane requires Rab1 and Rab8

**Figure 4:**
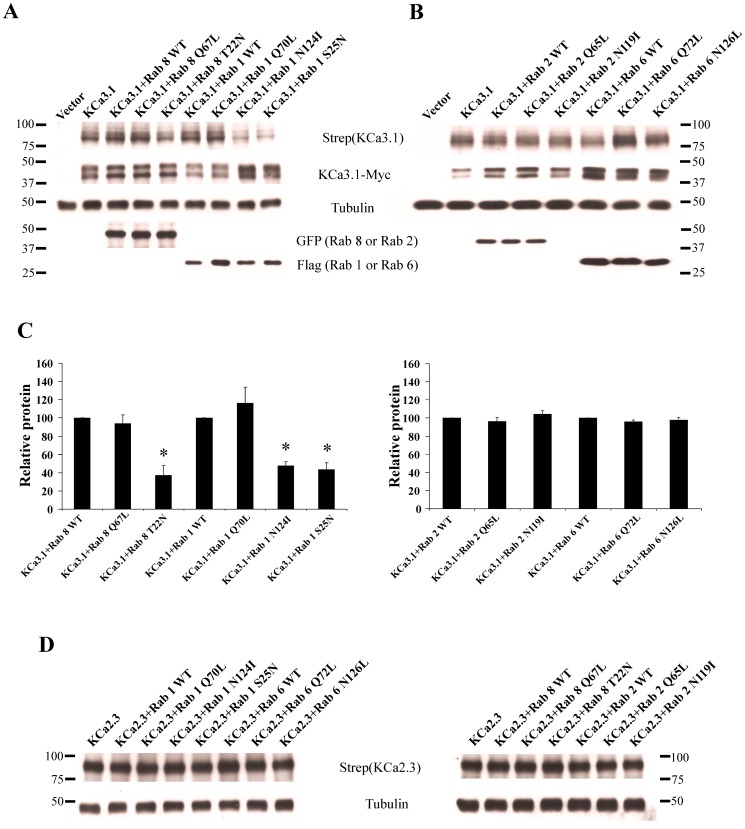
Plasma membrane localized KCa3.1 is decreased by DN Rab1 and Rab8, but not by Rab2 or Rab6. HEK293 cells were co-transfected with BLAP-KCa3.1 and either WT, DA or DN Rabs 1, 2, 6 or 8 and plasma membrane localized KCa3.1 evaluated by specific biotinylation, streptavidin labeling and IB (see Methods). As shown in **A** and **C**, DN Rab8 (T22N) and two different DN Rab1s (N124I, S25N) decreased expression of plasma membrane KCa3.1. In contrast, DN Rab2 and Rab6 had no effect on membrane expression of KCa3.1 (**B** and **C**). 30 μg of protein was loaded per lane. The bar graphs in **C** are averages of 3 experiments; *P<0.05 with respect to WT Rabs. **D**. HEK293 cells were co-transfected with BLAP-KCa2.3 and either WT, DA or DN Rabs 1, 2, 6 or 8 and membrane localized KCa2.3 evaluated as above. None of these Rabs had any effect on membrane expression of KCa2.3 (n = 3).

Rabs 1, 2 and 6 have been shown to be involved in the trafficking of proteins from the ER to Golgi, whereas Rabs 8 and 10 are known to be involved in the trafficking of protein from the Golgi to the plasma membrane [26], [27], [32], [33]. We initially determined whether these Rabs are involved in the trafficking of KCa3.1 to the plasma membrane in HEK293 cells. For these studies, we co-transfected BirA-KDEL/BLAP-KCa3.1 with either WT, DA or DN forms of each of these Rabs such that BLAP-KCa3.1 would be biotinylated in the ER. Plasma membrane BLAP-KCa3.1 was then detected by streptavidin labeling and IB, as above. We evaluated total KCa3.1 expression by co-transfecting a myc-tagged KCa3.1 with the relevant Rabs and blotted for myc. As shown in Figs. 4A and B, while WT or DA forms of Rab1 (Q70L) and Rab 8 (Q67L) did not affect plasma membrane expression of KCa3.1, dominant negative Rab8 (T22N: 37±10%, n = 3; P<0.05) and Rab1 (N124I: 48±4%; S25N: 44±8%; n = 3; P<0.05) significantly decreased KCa3.1 plasma membrane expression. In contrast, we did not observe any effect of Rabs 2 or 6 on plasma membrane expression of KCa3.1 (Fig. 4B). Similarly, we observed no effect of Rab 10 on plasma membrane expression of KCa3.1 (data not shown). We also determined whether the effects of the Rabs observed were specific for KCa3.1 or whether the plasma membrane expression of another family member, KCa2.3, would be similarly affected. As shown in Fig. 4D, Rabs 1, 2, 6 or 8 had no effect on plasma membrane expression of KCa2.3.

**Figure 5:**
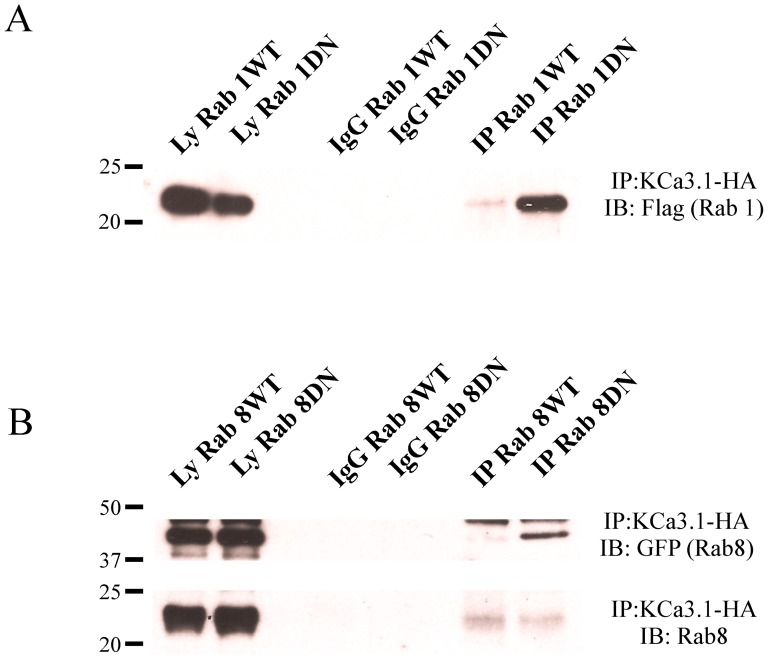
KCa3.1 immunoprecipitates with Rab1 and Rab8. **A**. Co-IP of HA-tagged KCa3.1 and either WT or DN Rab1 (Flag) was carried out in HEK293 cells as described in the Methods. Total cell lysates were subject to IP using either an α-HA Ab (lanes 5, 6) or an α-V5 Ab as IgG control (lanes 3, 4) and subsequently blotted for Rab1 (α-Flag). **B**. Co-IP of HA-tagged KCa3.1 and either endogenous (bottom panel) or GFP-tagged WT and DN Rab8 (top panel). Total cell lysates were subject to IP as in A and subsequently IB for either endogenous Rab8 (α-Rab8) or GFP-tagged Rab8 (α-GFP). Lysates for WT and DN Rab1 or 8 are shown in the first two lanes of each blot (5 μg loaded per lane). These data confirm an association between KCa3.1 and Rabs1 and 8. Data are representative of 3 experiments.

We also carried out co-IP experiments to determine whether KCa3.1 is associated with Rab1 WT/DN or Rab8 WT/DN. Thus, we co-transfected HA-tagged KCa3.1 with either Flag-tagged Rab1 or GFP-tagged Rab8 in to HEK293 cells and immunoprecipitated with α-HA antibody followed by IB for the appropriate heterologously expressed Rab. As shown in Figs. 5A and B, KCa3.1 co-immunoprecipitated predominantly with the DN forms of Rab1 and Rab8. Additionally, in our Rab8 experiments we were able to directly assess interactions between KCa3.1 and endogenous Rab8 by blotting using an αRab8 Ab. As shown in Fig. 5B (lower panel), we were able to co-IP endogenous Rab8 with KCa3.1 in the presence of heterologously expressed WT and DN Rab8. Indeed, we observed similar levels of endogenous Rab8 in both conditions, indicating that some Rab8 continues to associate with KCa3.1 and promote forward trafficking of the channel in the presence of DN Rab8.

**Figure 6:**
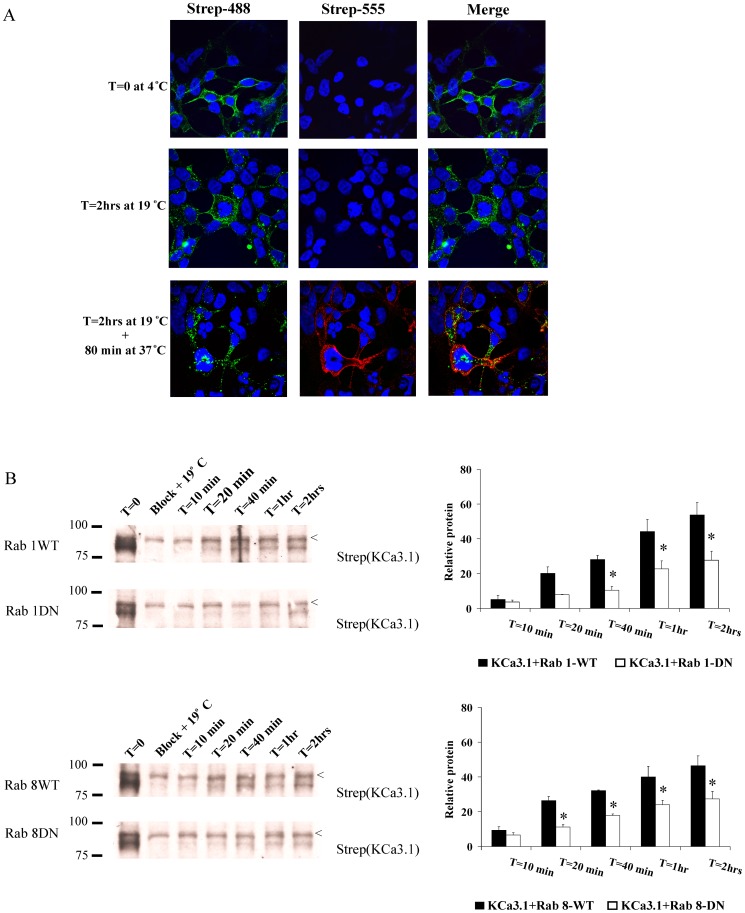
DN Rab1 and Rab8 slow ER/Golgi-to-plasma membrane delivery of KCa3.1. **A**. IF illustration of plasma membrane BLAP-KCa3.1 blocking protocol and subsequent appearance of new BLAP-KCa3.1 to the plasma membrane along the biosynthetic pathway. HEK293 cells were transfected with BLAP-KCa3.1 and BirA-KDEL in the pBudCE_4.1_ plasmid such that BLAP-KCa3.1 is specifically biotinylated at the level of the ER (see Methods). **Top row**: Plasma membrane BLAP-KCa3.1 was labeled with strepatavidin-Alexa488 (left; green) and subsequently with streptavidin-Alexa555 (middle; red). As is apparent, strep-488 saturated the biotin binding sites, precluding strep-555 labeling; demonstrating block of existing plasma membrane localized KCa3.1 channels. **Middle row**: Following 2 hrs at 19°C to accumulate KCa3.1 in the Golgi, some of the channels initially labeled at the plasma membrane (strep-488) have been endocytosed. Labeling with strep-555 demonstrates that no new channels have trafficked to the plasma membrane during this 19°C incubation, as expected. **Bottom row**: Further incubation at 37°C for 80 min results in endocytosis of KCa3.1 channels initially labeled with strep-488 at T = 0. Labeling with strep-555 demonstrates that new KCa3.1 channels have appeared at the plasma membrane, consistent with trafficking out of the Golgi. Nuclei are labeled with DAPI (blue). **B**. Experiments were carried out as illustrated in A except that blocking of existing plasma membrane KCa3.1 was carried out using neutravidin. As shown in lane 2 for each blot, neutravidin eliminated greater than 90% of the labeling attributed to streptavidin binding to plasma membrane BLAP-KCa3.1. Within 10 min incubation at 37°C, KCa3.1 begins to appear at the plasma membrane following ER/Golgi exit. After 2 hrs, KCa3.1 has recovered ∼50% relative to T = 0 in the presence of WT Rab1 and Rab8, whereas this is decreased to ∼27% in the presence of DN Rab1 and Rab8. 30 μg of protein was loaded per lane. The average of 3 experiments is shown in the bar graphs. The solid bars indicate expression of KCa3.1 in the presence of WT Rab while the open bars show expression of KCa3.1 in the presence of DN Rab (* P<0.05 with respect to same period of time with WT Rab). “<” indicates a non-specific band. These results indicate that DN Rab1 and Rab8 slow exit of KCa3.1 from the ER/Golgi resulting in decreased plasma membrane expression as suggested in Fig. 4.

If the expression of a DN Rab is slowing the exit of KCa3.1 from the ER/Golgi this should be apparent as a decreased rate of trafficking to the plasma membrane. Our method for defining this step is shown by IF in Fig. 6A. For these studies, we expressed BirA-KDEL/BLAP-KCa3.1 in HEK293 cells. Expression of BLAP-KCa3.1 at the plasma membrane is confirmed by streptavidin-Alexa488 labeling (T = 0 at 4°C). Immediately labeling with streptavidin-Alexa555 results in no signal (T = 0 at 4°C; Strep-555), confirming that the biotin sites on KCa3.1 are saturated or “blocked” by streptavidin-Alexa488 binding. The cells are then incubated at 19°C for 2 hrs to accumulate BLAP-KCa3.1 in the ER/Golgi (T = 2 hrs at 19°C). As is apparent, some of the streptavidin-Alexa488 channel has been endocytosed during this 19°C incubation step, however, no additional channel has trafficked to the plasma membrane as assessed by the lack of streptavidin-Alexa555 labeling. Following incubation of the cells at 37°C for 80 min, we observed small green puncta inside the cell consistent with endocytosis of plasma membrane KCa3.1. In addition, new channels have trafficked to the plasma membrane from the ER/Golgi such that they can be “captured” as is apparent by our ability to label these newly arrived channels with streptavidin-Alexa555. To quantify this rate, we utilized neutravidin to “block” existing BLAP-KCa3.1 channels at the plasma membrane and “captured” newly arrived channels with streptavidin followed by IB, as above. The amount of channel “captured” at any time point was then compared to the amount of channel present at T = 0, which represents steady-state expression levels. Any signal left following “block” was subtracted from all subsequent time points as this represented the amount of channel that could not be bound by neutravidin. As shown in Fig. 6B, KCa3.1 rapidly exits the ER/Golgi and begins to appear at the plasma membrane within 10 min and the level of channel expression continues to increase over time. In the presence of WT Rab1 or Rab8 after 2 hrs at 37°C expression levels of KCa3.l at the plasma membrane had returned to 54±7% and 46±5% of those seen at T = 0, respectively. In contrast, in the presence of DN Rab1 and Rab8 this was only 28±7 (n = 3; P<0.05) and 27±4% (n = 3; P<0.05), respectively, indicating that expression of DN Rab1 and Rab8 decreases the rate of KCa3.1 trafficking from the ER/Golgi to the plasma membrane.

**Figure 7:**
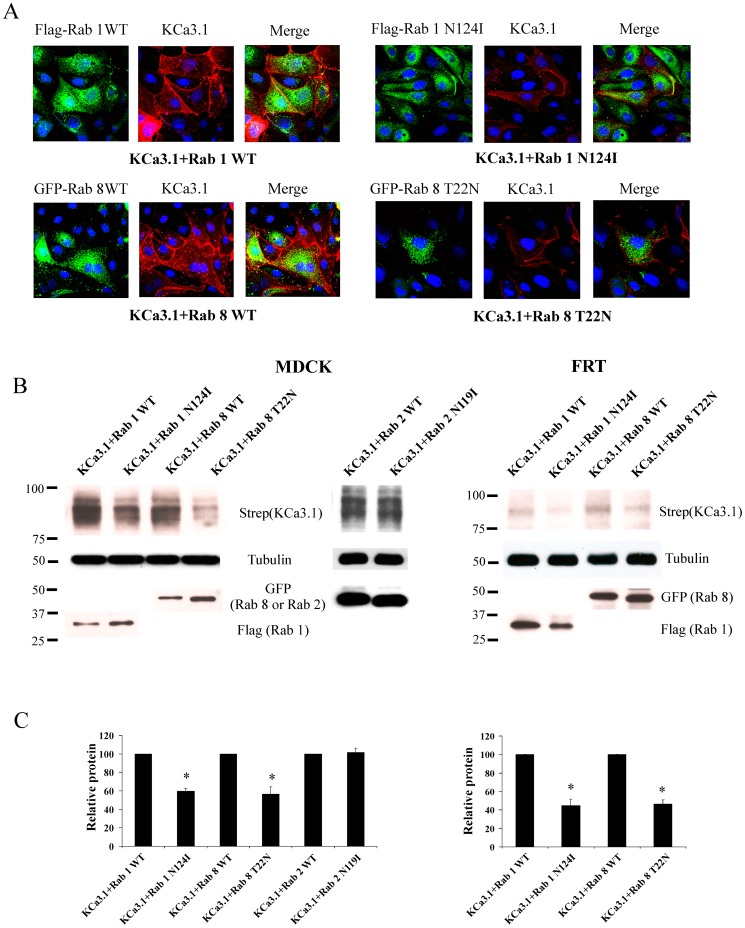
DN Rab1 and Rab8 decrease plasma membrane expression of KCa3.1 in polarized epithelia. **A**. BLAP-KCa3.1 and either WT or DN Flag-Rab1 (N124I) (top row) or GFP-Rab8 (T22N) (bottom row) were transfected into confluent MDCK cells. Plasma membrane KCa3.1 was labeled with streptavidin-Alexa555 (red) after which the cells were fixed/permeabilized and Rab1 labeled with α-Flag Ab (Rab8 is GFP tagged). Both DN Rab1 (top panels) and Rab8 (bottom panels) result in an apparent decrease in plasma membrane KCa3.1 expression. Nuclei are labeled with DAPI (blue). **B**. BLAP-KCa3.1 and either WT or DN Flag-Rab1 (N124I) or GFP-Rab8 (T22N) were transfected into either MDCK (left) or FRT (right) cells and plasma membrane KCa3.1 evaluated by IB (see Methods). WT and DN Rab2 were transfected in to MDCK cells together with BLAP-KCa3.1 as a negative control based on our HEK298 data (Fig. 4). 10 μg of protein was loaded per lane. Tubulin was used as a loading control. Expression of Rab1 and Rab8 were confirmed by IB using α-Flag and α-GFP Ab, respectively. **C**. Quantification of 3 separate blots demonstrates that DN Rabs1 and 8 decrease plasma membrane expression of KCa3.1 in both MDCK and FRT cells (*P<0.05 with respect to WT Rabs), whereas DN Rab2 had no effect on membrane KCa3.1 expression in MDCK cells.

We further determined whether Rabs 1 and 8 are involved in the trafficking of KCa3.1 to the plasma membrane in polarized MDCK and FRT cells. Initially, we carried out IF studies by transducing BirA-KDEL/BLAP-KCa3.1 and transfecting either WT or DN Flag-Rab1 or GFP-Rab8. BLAP-KCa3.1 was labeled at the plasma membrane with streptavidin-Alexa555 and Flag-Rab1 was labeled with α-Flag Ab following fixation/permeabilization; GFP-Rab8 was directly visualized. As shown in Fig. 7A, expression of either DN Rab1 (N124I) or Rab8 (T22N) resulted in an apparent decrease in plasma membrane expression of KCa3.1. To confirm this result biochemically, we carried out IB of plasma membrane BLAP-KCa3.1. As shown in Figs. 7B and C, both DN Rab1 and Rab8 resulted in a significant decrease in plasma membrane expression of KCa3.1 in both MDCK and FRT cells. In 3 experiments, Rab1 N124I decreased plasma membrane expression to 60±3% and 45±7% (P<0.05) of that observed following WT Rab1 expression in MDCK and FRT cells, respectively (Fig. 7C). Similarly, Rab8 T22N decreased plasma membrane expression to 57±8% and 47±4% (P<0.05) of that observed following WT Rab8 expression in MDCK and FRT cells, respectively (Fig. 7C). Similar to our HEK293 data, DN Rab2 had no effect on plasma membrane KCa3.1 expression in MDCK cells (Fig. 7B).

### The AP-1 adaptor protein, μ1B is not required for proper BL sorting of KCa3.1 in polarized epithelia

**Figure 8:**
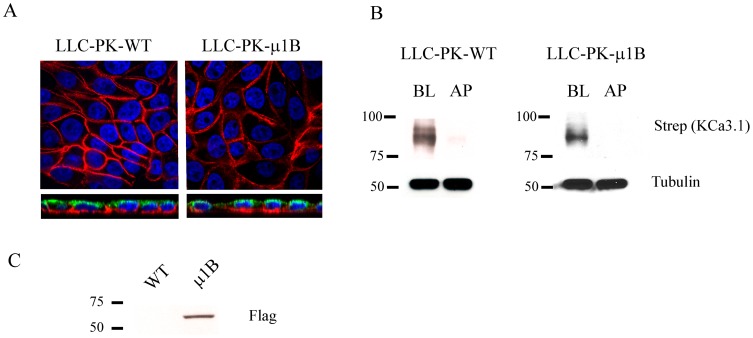
Basolateral targeting of KCa3.1 is independent of the adaptor protein, μ1B. **A**. BLAP-KCa3.1 was transduced in to either WT LLC-PK_1_ cells (left panels) or LLC-PK_1_ cells stably expressing μ1B (right panels) grown to confluence on Transwell® filters. The AP and BL membranes were biotinylated using recombinant BirA and biotinylated proteins labeled with streptavidin-Alexa555 (red). Apical membrane was co-labeled with WGA-Alexa488 (green). Nuclei were labeled with DAPI (blue). The top panels show a single confocal section through the mid-plane of the cells and the bottom panels show a z-stack. KCa3.1 was detected exclusively in the BL of both LLC-PK_1_ clones. **B**. Either AP or BL membranes of WT LLC-PK_1_ cells (left panel) or LLC-PK_1_ cells stably expressing μ1B (right panel) grown to confluence on Transwell® filters were biotinylated using BirA followed by streptavidin labeling and subsequent IB to determine localization of KCa3.1. KCa3.1 was localized specifically to the BL membrane in both LLC-PK1 clones. Tubulin was used as a loading control. Blots are representative of 3 separate experiments. 20 μg of protein was loaded per lane. **C**. IB confirming expression of Flag-tagged μ1B in the LLC-PK_1_-μ1B cell line.

It has been shown that the μ1B subunit of the adaptor protein complex AP-1 is important in the BL sorting of several proteins in polarized epithelia [34]. Importantly, while μ1B is expressed in MDCK cells it is not expressed in LLC-PK_1_ cells resulting in the mis-targeting of some BL proteins [35]. To determine whether μ1B was required for BL targeting of KCa3.1 we transduced BLAP-KCa3.1 in to both parental and μ1B-Flag corrected LLC-PK_1_ cells and evaluated BL vs apical localization by both IF and IB, as above. As shown in Fig. 8, KCa3.1 was targeted to the BL membrane in both LLC-PK_1_-WT and μ1B-expressing LLC-PK_1_ cells based upon both IF (A) and IB (B). Expression of μ1B was confirmed by IB (Fig. 8C). These data indicate that the BL sorting of KCa3.1 in polarized epithelia is independent of the μ1B subunit of AP-1 complex.

### KCa3.1 traffics directly to the basolateral plasma membrane in polarized epithelia

**Figure 9:**
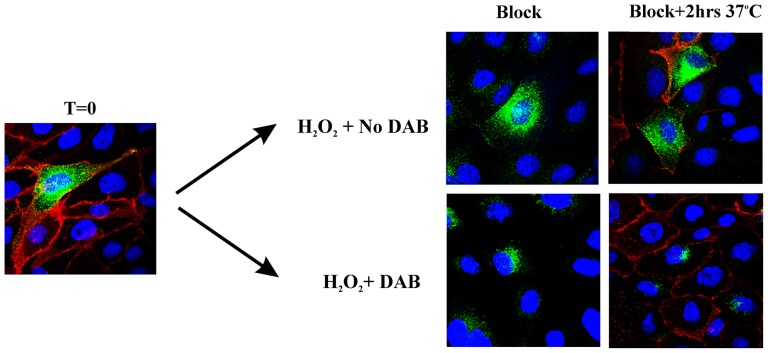
KCa3.1 does not traffic via transferrin-positive recycling endosomes to the plasma membrane in MDCK cells. MDCK cells were transduced with Transferrin receptor (TfnR) and BirA-KDEL/BLAP-KCa3.1 after which the TfnR was bound to a mixture of Tfn-Alexa488 (green) and Tfn-HRP and allowed to endocytose for 40 min at 37°C. Plasma membrane BLAP-KCa3.1 was then labeled with streptavidin-Alexa555 (red). This is shown in the left panel (T = 0) and confirms that under these conditions we are able to label both TfnR and KCa3.1 in confluent MDCK cells. To determine whether KCa3.1 traffics to the plasma membrane via TfnR-positive recycling endosomes, we labeled the TfnR as above and blocked plasma membrane BLAP-KCa3.1 with neutravidin. Subsequently, recycling endosome ablation was carried out by reacting the Tfn-HRP with H_2_O_2_ + DAB (DAB was omitted as a non-ablation control) and ER/Golgi exit allowed to proceed for 2 hrs at 37°C after which KCa3.1 that was newly trafficked to the plasma membrane was labeled with strep-555 (see Methods and Results sections for additional details). As shown in the T = 0 panel and the no DAB control (top right panels), Tfn-488 is expressed in endosomes throughout the cytoplasm as it recycles to the plasma membrane, as expected. However, following DAB-dependent ablation, Tfn-488 is clustered in the recycling endosomes as evidenced by their perinuclear localization; confirming recycling endosome ablation resulting in inhibition of TfnR trafficking back to the plasma membrane. Importantly, in both ablated and non-ablated cells, BLAP-KCa3.1 traffics to the plasma membrane demonstrating that KCa3.1 does not traffic through TfnR-positive recycling endosomes on the way to the plasma membrane. Images are representative of 3 separate experiments.

It has been shown that proteins destined for the BL membrane can traffic either: 1) from the Golgi to the BL membrane directly or 2) from the Golgi through recycling endosomes to the BL membrane [36], [37]. To determine whether KCa3.1 traffics via recycling endosomes we carried out recycling endosome ablation in MDCK cells [29]. We transduced MDCK cells with both transferrin receptor (TfnR) and BirA-KDEL/BLAP-KCa3.1 adenoviruses. Initially, both Tfn-HRP and Tfn-Alexa488 were bound to TfnR and allowed to internalize for 45 min at 37°C, thereby allowing the TfnR with bound substrate to traffic through early and recycling endosomes. Subsequently, BLAP-KCa3.1 was labeled with either streptavidin-Alexa555, to confirm expression of the channel at the plasma membrane at T = 0, or neutravidin to “Block” plasma membrane resident channels (see also, Fig. 6). As shown in Fig. 9, at T = 0 Tfn-Alexa488 was located in endosomal compartments, as is apparent from the green puncta, whereas KCa3.1 is localized to the plasma membrane. Next, recycling endosome ablation was carried out by treating the cells with DAB, in the absence (control) or presence (ablation) of H_2_O_2_. As is apparent in the images labeled “Block”, in the absence of DAB the Tfn-Alexa488 is located in vesicles throughout the cytoplasm, whereas in the presence of DAB the Tfn-Alexa488 is localized exclusively to perinuclear recycling endosomes, as reported [29]. These results confirm recycling endosome ablation such that TfnR can no longer exit from these vesicles. Also, BLAP-KCa3.1 could not be labeled by streptavidin-Alexa555, confirming “Block” by neutravidin. Finally, the cells were returned to 37°C for 2 hrs in the presence of cyclohexamide such that KCa3.1 could exit the Golgi and traffic to the plasma membrane after which these channels were labeled with streptavidin-Alexa555. As shown (Block +2 hrs 37°C), KCa3.1 trafficked to the plasma membrane in both the control and recycling endosome ablated cells, consistent with KCa3.1 trafficking directly to the plasma membrane rather than via the TfnR-positive recycling endosomes.

**Figure 10:**
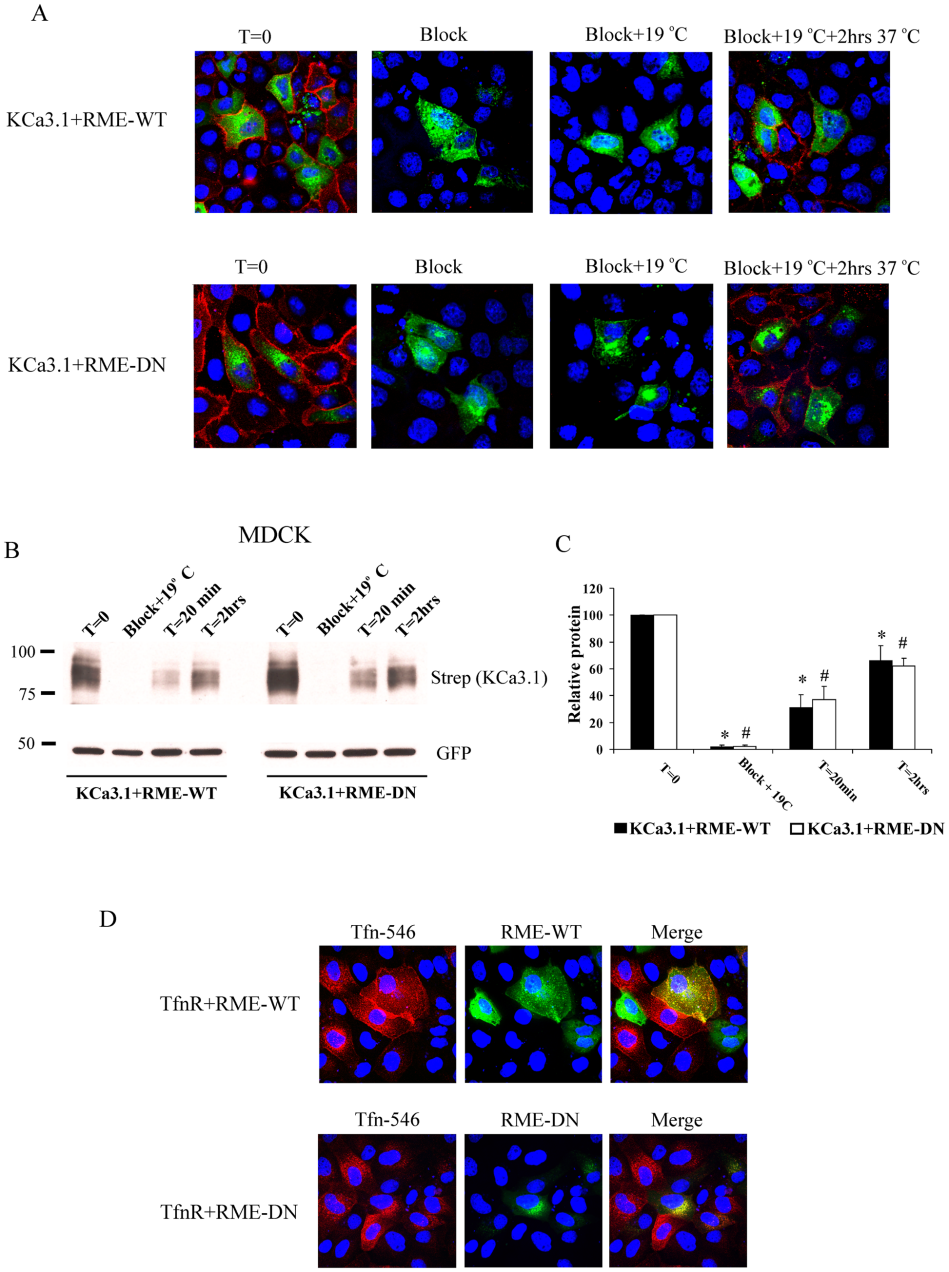
KCa3.1 does not traffic to the plasma membrane via RME-1-positive recycling endosomes. **A**. MDCK cells were transfected with BLAP-KCa3.1 and either GFP-tagged WT (top panels) or DN (bottom panels) RME-1. Plasma membrane localized KCa3.1 could be labeled with streptavidin-Alexa555 (T = 0) following biotinylation and this could be blocked by prior incubation with neutravidin (Block). Following block and ER/Golgi trap at 19°C BLAP-KCa3.1 is not detected at the plasma membrane (Block + 19°C). Subsequent incubation at 37°C for 2 hrs results in the appearance of BLAP-KCa3.1 at the plasma membrane in both WT- and DN-RME-1-expressing MDCK cells. **B**. Experiments in A were quantified by IB. As is apparent, at both 20 min and 2 hrs the rate of appearance of KCa3.1 at the plasma membrane is not altered by DN RME-1 expression. 10 μg of protein was loaded per lane. WT and DN RME-1 expression was confirmed by IB using α-GFP Ab. The results of 3 separate experiments are quantified in **C**. The filled bars indicate expression of KCa3.1 in the presence of WT RME-1 (*P<0.05 with respect to T = 0), whereas the open bars indicate expression of KCa3.1 in the presence of DN RME-1 (#P<0.05 with respect to T = 0) (n  =  3). No differences in the rate of KCa3.1 plasma membrane appearance were seen indicating KCa3.1 does not traffic through RME-1-positive recycling endosomes on the way to the plasma membrane. **D**. MDCK cells were transduced with TfnR and transfected with GFP-tagged WT (top panels) and DN (bottom panels) RME-1. TfnR was bound to Tfn-Alexa546 and allowed to endocytose at 37°C for 45 min after which the cells were fixed/permeabilized (see Methods). In the presence of WT RME-1, Tfn-546 is distributed evenly throughout the cytoplasm, whereas in the presence of DN RME-1 Tfn-546 is trapped in a perinuclear compartment. Note that in cells not expressing the DN RME-1, Tfn-546 is localized throughout the cytoplasm similar to what is observed with WT RME-1 expression. These results demonstrate that, in contrast to KCa3.1, the TfnR traffics through RME-1-positive recycling endosomes.

As an alternate strategy to disrupt recycling endosomes we co-expressed either WT or DN RME-1 (G429R) with BLAP-KCa3.1. DN RME-1 induces redistribution of recycling endosomes and inhibits the exit of proteins such that cargo delivered to the DN RME-1 positive endosomes becomes trapped in this compartment [38]. Thus, we determined whether expression of G429R-RME-1 would inhibit delivery of KCa3.1 to the plasma membrane following Golgi exit. MDCK were transduced with BirA-KDEL/BLAP-KCa3.1 adenovirus and transfected with WT or DN GFP-tagged RME-1. As shown in Fig. 10A, at T = 0, KCa3.1 was labeled with strepavidin-Alexa555 at the plasma membrane in the presence of WT or DN RME-1 and this labeling could be blocked by neutravidin (Block). Following incubation at 19°C to accumulate KCa3.1 in the Golgi, we were unable to detect KCa3.1 in the plasma membrane (Block+19°C), as above (Figs. 6 and 9). Finally, the MDCK cells were returned to 37°C for 2 hrs to allow channels to exit the Golgi and traffic to the plasma membrane after which the channel was labeled with strepavidin-Alexa555 (Block+19°C+2 hrs 37°C). As is apparent, in both WT and DN RME-1-expressing MDCK cells, KCa3.1 trafficked to the plasma membrane. We confirmed this result by IB by determining whether the rate of KCa3.1 appearance at the plasma membrane was altered by DN RME-1 expression. As shown Figs. 10B and C, blocking with neutravidin eliminated the signal associated with plasma membrane KCa3.1. After Golgi release at 37°C for 20 min KCa3.1 is clearly apparent at the plasma membrane and this expression increases after 2 hrs. Importantly, similar levels of KCa3.1 were expressed at the plasma membrane in MDCK cells expressing either WT or G429R RME-1. In 3 experiments, after 2 hrs at 37°C there was no difference in expression of KCa3.1 at the plasma membrane, being 66±11% of control (T = 0) in WT RME-1 expressing cells and 62±6% of control (T = 0) in G429R RME-1 expressing cells (Fig. 10C). These results further demonstrate that KCa3.1 traffics directly from the Golgi to the BL membrane rather than indirectly via recycling endosomes.

Finally, we determined whether TfnR would be trapped in RME-1-positive endosomes by transducing MDCK cells with TfnR followed by transfection with either WT or DN GFP-tagged RME-1. TfnR was bound to Tfn-Alexa546 and allowed to endocytose for 45 min at 37°C, after which the cells were fixed for IF localization. As shown in Fig. 10D, in the presence of WT RME-1, the TfnR is in endosomes throughout the cytoplasm. However, in the presence of DN RME-1, the TfnR is clustered in the RME-1-positive compartment. This result confirms that DN RME-1 is capable of trapping proteins that traffic via recycling endosomes.

## Discussion

It is now clear, based on both electrophysiological and pharmacological data, that KCa3.1 is expressed in the BL membrane of numerous epithelia [6], [7], [8], [9]. However, more recent evidence has demonstrated K^+^ secretion in both primary cultures of human bronchial epithelium [12] and proximal colon [39] that is sensitive to block by apical charybdotoxin and clotrimazole; indicative of KCa3.1 channels in the apical membrane. Based on these data, Rajendran and colleagues [40] identified a unique transcript, KCNN4c, that lacks exon 2 which encodes for the second transmembrane domain of KCa3.1. These authors demonstrated that this channel targeted to the apical membrane when co-assembled with the large conductance, Ca^2+^-activated K^+^ channel β-subunit [40]. While these data demonstrate that KCa3.1 can be targeted to either the apical or BL membrane of polarized epithelia, depending on the transcript expressed, there is no information to date demonstrating how this channel is targeted to the BL membrane or its' endocytic fate once it is in the BL membrane. Herein, we have used the BLAP-tagged KCa3.1 to begin to address these questions. We demonstrate that KCa3.1-BLAP is highly expressed at the BL membrane with insignificant expression in the apical membrane of three different polarized epithelial cell models, MDCK, Caco-2 and FRT (Fig. 1A). Similar to what we previously reported in HEK293 cells [22], KCa3.1 was degraded with a half-time of ∼3-5 hrs in MDCK and FRT cells. However, in Caco-2 cells the half-time for degradation was significantly longer, being ∼16 hrs (Fig. 1B). At present it is unclear why the degradation of KCa3.1 is slowed in Caco-2 cells. While this may suggest that KCa3.1 degradation is uniquely regulated in intestinal epithelia, more studies are required to determine this.

We demonstrate that, following endocytosis from the BL membrane of polarized epithelia, degradation is blocked by inhibiting lysosomal proteases (Fig. 2); similar to what we previously reported in HEK293 cells [19]. While we have demonstrated a role for p97/Derlin-1 and the proteasome in the degradation of mis-folded KCa3.1 channels in the ER [17] a role for the proteasome in the degradation of KCa3.1 following endocytosis has not been investigated. As shown in Fig. 2, lactacystin inhibited KCa3.1 degradation, indicative of a role for the 26 S proteasome in targeting KCa3.1 to the lysosome for degradation. Numerous studies have shown that the degradation of membrane resident proteins is also blocked by inhibitors of the proteasome [41], [42], [43]. In this regard, it has been suggested that the ubiquitin-proteasome pathway is involved in the endosomal sorting step of some proteins to the lysosome [44]. More recently, it was shown that Ecm29 binds the 26 S proteasome and links the proteasome to various endocytic proteins, including Rab11 and rabaptin via its N-terminal end and molecular motors via its C-terminal end [45]. Also, proteasomal inhibition by MG132 for 20 hrs caused redistribution of the endosomal adaptor protein, APPL1 as well as the early endosomal marker, Rab5 to the perinuclear region of cells, whereas the early endosomal marker, EEA1 was unaffected [46]. Thus, the 26 S proteasome may be directly coupled to KCa3.1-containing endosomes, thereby influencing its' degradation.

To date, more than 60 Rabs have been identified and it is thought that each Rab is related with a particular organelle or pathway [32], [47]. Rab 2 has been shown to be predominantly localized to the ER/Golgi intermediate compartment (ERGIC) where it is involved in the retrograde transport from the ERGIC to the ER [48]. Rab 6 is mainly localized to the Golgi and the trans-Golgi network (TGN) where it modulates the retrograde transport between the Golgi cisternae or from the Golgi to the ER [25]. Expression of DN Rab 2 and Rab 6 have been shown to inhibit the anterograde transport of numerous proteins, including the cystic fibrosis transmembrane conductance regulator (CFTR) and G-protein coupled receptors (GPCR) [48], [49]. Also, Rab 1 is localized in the ER and the Golgi where it regulates anterograde transport from the ER to the Golgi and among the Golgi compartments [32]. The manipulation of Rab1 function has been shown to block the ER to cell surface transport of α-adrenergic, angiotensin II type 1 and human calcium sensing receptors [50], [51], [52]. Finally, Rab 8 and Rab 10 have been extensively investigated in polarized epithelial cells where they have been shown to play a role in protein trafficking from the Golgi to the BL membrane [27], [33], [53]. Herein, we demonstrate that Rab1 and Rab8 play a crucial role in the anterograde trafficking of KCa3.1 to the BL membrane of polarized MDCK cells (Fig. 7), whereas Rabs 2, 6 and 10 appear to play no role in KCa3.1 trafficking. Indeed, we demonstrate that expression of DN Rab1 or Rab 8 slowed the appearance of KCa3.1 to the plasma membrane in HEK cells (Fig. 6), consistent with their known roles in the forward trafficking of various proteins [27], [50], [51]. Interestingly, a closely related family member, KCa2.3 was unaffected by expression of DN forms of any of these Rabs (Fig. 4D), indicating a high degree of specificity amongst these KCa family members. Unfortunately, chimeras between KCa3.1 and KCa2.3 did not reveal any apparent domains critical for this Rab-dependent trafficking of KCa3.1 (C.A. Bertuccio and D.C. Devor, unpublished observations).

GPCR have been shown to directly interact with members of the Rab family, including Rab1 and Rab8 which interact with β_2_- and α_2_- adrenergic receptors [27], [54]. Herein, we demonstrate a close association between KCa3.1 and WT or DN Rab1 and Rab8 by co-IP (Fig. 5). Interestingly, KCa3.1 preferentially associates with the inactive, GDP-bound form of Rab1 and Rab8, similar to what has been described for some GPCR [27], [54]. Indeed, it has been suggested that these receptors can interact with and activate Rab GTPases, thus facilitating their own transport [27], [55], although this is not known for KCa3.1.

Sorting of numerous proteins to the BL domain of polarized epithelia is the result of an association with the μ1 subunit of the adaptor protein-1 complex (AP-1) [56], [57]. The AP-1 complex is the only clathrin-associated adaptor complex implicated in BL sorting in polarized epithelia [58]. Importantly, while μ1B is expressed in MDCK and Caco-2 cells it is not expressed in LLC-PK_1_ cells. Lack of the μ1B subunit in LLC-PK_1_ cells has been shown to result in the mistargeting of some BL proteins to the apical surface and stable expression of μ1B restores proper BL targeting [35]. In addition, Ang et al. demonstrated that Rab8 acts in conjunction with AP-1B to target proteins to the BL membrane of polarized epithelia [53]. Since KCa3.1 is endogenously expressed in MDCK cells [59], is endocytosed in a clathrin-dependent manner [21] and its plasma membrane expression is modulated by Rab8 (Figs. 4 and 7), we evaluated whether μ1B was required for BL targeting of KCa3.1 using both LLC-PK_1_-WT and μ1B-expressing LLC-PK_1_ cells. As shown in Fig. 8, KCa3.1 was targeted to the BL membrane in both WT and μ1B-expressing LLC-PK_1_ cells, indicating that the BL sorting of KCa3.1 in polarized epithelia is independent of the μ1B subunit of the AP-1 complex. Similarly, Caplan and colleagues demonstrated that the sorting of the H^+^/K^+^-ATPase and Na^+^/K^+^-ATPase were independent of μ1B expression in polarized epithelia [28], [60].

As noted, proteins trafficking to the plasma membrane along the biosynthetic route, in both polarized and non-polarized cells, can either traffic directly from the Golgi to the plasma membrane or they can transit via recycling endosomes [29], [32], [37], [61], [62]. To determine whether KCa3.1 traffics through recycling endosomes before arriving at the plasma membrane, we monitored KCa3.1 appearance at the plasma membrane using two separate strategies. First, we carried out recycling endosome ablation using Tfn-HRP which resulted in the TfnR being trapped in a perinuclear compartment (Fig. 9) [29]. However, KCa3.1 trafficked to the BL membrane, indicating this channel does not traffic through TfnR-positive recycling endosomes. Second, we utilized a DN RME-1 approach to disrupt recycling endosomes. Lin et al. [38] showed that a mutation in the epsin homology domain (G429R) induced redistribution of recycling endosomes and inhibits the exit of proteins from this compartment. We demonstrated that, following endocytosis, KCa2.3 is targeted to this RME-1-positive compartment, whereas KCa3.1 was not [22]. In Fig. 10, we show that KCa3.1 is similarly not targeted to recycling endosomes following Golgi exit along the biosynthetic route. Indeed, we demonstrate that the rate of KCa3.1 appearance at the plasma membrane is not altered by DN RME-1 expression (Fig. 10B), suggesting no alteration in the rate of delivery. Thus, using two separate methods of disrupting the recycling endosome compartment demonstrates that KCa3.1 does not traffic through this compartment along the biosynthetic route.

The regulated trafficking of proteins to the BL membrane is an important process that controls the amount of protein at the cell surface and thereby modulates their function. Herein, we demonstrate that KCa3.1 is targeted to the BL membrane of polarized epithelia in a Rab1- and Rab8-dependent manner. However, following Golgi exit the correct BL targeting of KCa3.1 is independent of the AP-1B complex. In addition, we demonstrate that the trafficking itinerary of the newly synthesized KCa3.1 channels does not involve passage through the recycling endosome compartment. Finally, following correct insertion in to the BL membrane, KCa3.1 is endocytosed and targeted for lysosomal degradation in a process that requires initial ubiquitylation followed by de-ubiquitylation. Inhibition of this process by lactacystin indicates that proteasomes are functionally associated with KCa3.1-containing endosomes.
